# Lived Experiences of School-Age Children with Food Allergies: A Qualitative Systematic Review and Meta-Synthesis

**DOI:** 10.3390/children12081053

**Published:** 2025-08-11

**Authors:** Noriko Nishida, Yuki Maeda, Ikuo Okafuji, Shingo Ueki

**Affiliations:** 1 School of Nursing, Faculty of Nursing, Osaka Aoyama University, 2-11-1 Niina, Minoh 562-8580, Osaka, Japan; 2 Division of Nursing, School of Medicine, Gifu University, 1-1 Yanagido, Gifu 501-1193, Gifu, Japan; maeda.yuki.y0@f.gifu-u.ac.jp; 3 Department of Pediatrics, Kobe City Medical Center General Hospital, 2-1-1 Minatojima Minamimachi, Chuo-ku, Kobe 650-0047, Hyogo, Japan; okafuji@kcho.jp; 4 The Japan Centre for Evidence Based Practice—A JBI Centre of Excellence, Osaka University, 1-7 Yamadaoka, Suita 565-0871, Osaka, Japan; ueki.shingo.388@m.kyushu-u.ac.jp; 5 Department of Health Sciences, Faculty of Medical Sciences, Kyushu University, 3-1-1 Maidashi, Higashi-ku, Fukuoka 812-8582, Fukuoka, Japan

**Keywords:** food allergy, child, experience, school age, qualitative synthesis

## Abstract

**Highlights:**

**What are the main findings?**

- Children with food allergies live with daily fear, social restrictions, emotional stress, isolation, teasing, and exclusion from peers, whereas peer communication can foster understanding and acceptance.
- Some children engage in oral immunotherapy or allergen reintroduction, leading to increased food choices, social inclusion, and reduced anxiety.

**What is the implication of the main finding?**

- Child-centered, emotionally supportive, and inclusive care is vital to these children’s wellbeing, involving families, schools, and healthcare providers.

**Abstract:**

**Background/Objectives**: School-age children with food allergies (FAs) face substantial psychosocial challenges. Herein, we aimed to synthesize the experiences of such children. **Methods**: A systematic review of qualitative studies was conducted using the Joanna Briggs Institute methodology. The protocol was registered in PROSPERO (CRD42022359854). A systematic search was conducted of eight databases. Thirteen studies met the inclusion criteria. The data were synthesized through meta-aggregation, and the confidence in the findings was assessed using the ConQual approach. **Results**: Seventy-three unequivocal findings were extracted and synthesized into three integrated findings. (1) Children with FAs live with daily fear, social restrictions, and emotional stress. To ensure their safety and foster self-management skills, the trusted adults in their lives must be well-informed about allergy management. (2) Children with FAs experience isolation, teasing, and exclusion from peers, whereas peer communication can foster understanding and acceptance. To address this, schools must promote peer empathy, provide allergy education, and build inclusive environments that empower children to express their needs safely. (3) Motivated by curiosity and personal goals, some children engage in oral immunotherapy or allergen reintroduction, leading to meaningful outcomes, such as increased food choices, social inclusion, and reduced anxiety. At the same time, they face emotional and physical burdens, highlighting the need for safety-focused, informed, supported care that considers both the benefits and burdens. **Conclusions**: This review highlights the need for child-centered, emotionally supportive, and inclusive care involving families, schools, and healthcare providers. However, the moderate ConQual score of the synthesized findings indicates that the recommendations should be considered with caution.

## 1. Introduction

Food allergies (FAs) affect up to 10% of the global population and are more commonly observed in children than they are in adults [1]. More than 40% of children with FAs often experience potentially life-threatening reactions and carry an epinephrine autoinjector [2]. In response to this growing public health concern, the 2025 European Academy of Allergy and Clinical Immunology (EAACI) guidelines emphasize a comprehensive management approach that includes dietary strategies, psychological support, emergency preparedness, and immunomodulatory therapies [3].

The primary-school-age years (6–12 years) represent a critical period in child development, during which peer relationships play a central role in the shaping of identity, personality, and social competence [4]. Chronic health conditions during this stage can disrupt these developmental processes. A recent cohort study revealed that children with chronic conditions are more likely to experience emotional and behavioral problems in adolescence, highlighting the need for early psychosocial support and long-term monitoring [5]. As chronic conditions, FAs have been demonstrated to have a profound psychosocial impact on affected children. Beyond the physical risks associated with allergen exposure, children with FAs frequently experience anxiety, depression, post-traumatic stress, and social isolation [6]. FA-related bullying [7], FA-related stigma [8], and reduced quality of life [9,10,11,12,13] have also been widely reported. An understanding of these experiences is crucial to the development of holistic care approaches that address both the physical and emotional well-being of children.

A preliminary search identified two qualitative systematic reviews; however, both were focused on adolescents aged 11 years and older [14,15]. In one mixed-methods systematic review, studies of children and adolescents aged 0–12 years were included, combining parental reports with children’s perspectives [16]. That study was methodologically distinct in that the researchers integrated both qualitative and quantitative research. However, parents tend to underestimate the impact of FAs on their child’s quality of life [17]. Moreover, as emphasized in the United Nations Convention on the Rights of the Child, children have the right to participate and have their views considered [18]. Furthermore, previous reviews were limited to the English literature only.

To address these gaps, we aimed to synthesize the qualitative research that directly explores the lived experiences of primary-school-age children (6–12 years) with FAs. By including publications in any language and focusing on children’s own narratives, we sought to provide a more comprehensive and globally relevant understanding of how FAs affect children’s daily lives, emotional well-being, and social development. Meta-aggregation was used to systematically integrate the findings and inform future clinical and psychosocial interventions. Our findings emphasize that child-centered, emotionally supportive, and inclusive care is vital to these children’s well-being, involving families, schools, and healthcare providers.

## 2. Materials and Methods

### 2.1. Methodological Guidelines

This systematic review was conducted in accordance with the Joanna Briggs Institute (JBI) methodology for systematic reviews of qualitative evidence [19]. The protocol for this review was registered in PROSPERO (CRD42022359854). The protocol for this review was prepared a priori. The complete protocol for this systematic review is available upon request.

### 2.2. Review Questions

The review question was as follows: How do primary-school-age children experience life with FAs?

### 2.3. Inclusion Criteria

#### 2.3.1. Participants

We included studies involving primary-school-age children living with FAs. For the purposes of this review, primary school age was operationally defined as 6–12 years. The parents and medical professionals who care for children with FAs were excluded from this review. Both medically confirmed diagnoses and parent-reported FAs were included without restrictions. We did not limit the inclusion of children based on the types and numbers of foods to which they were allergic. Children with FAs who also had asthma, atopic dermatitis, and allergic rhinitis—conditions commonly associated with allergies—were included. However, children with other co-existing conditions unrelated to allergies, such as autism, were excluded, as such conditions might have introduced additional factors that could have influenced the outcomes.

#### 2.3.2. Phenomena of Interest

The phenomena of interest were the children’s perceptions and attitudes towards FAs.

#### 2.3.3. Context

We included studies conducted in various contexts to capture a wide range of voices. The review was not restricted to any specific country or region.

#### 2.3.4. Types of Studies

This was a review of qualitative studies. The eligible study designs included phenomenology, grounded theory, and qualitative descriptive studies. We included mixed-methods studies if the qualitative results could be analyzed separately.

### 2.4. Data Sources and Search Strategy

Two researchers (NN and SU) and a medical librarian devised a search strategy to identify studies for this review. We aimed to find both published and unpublished studies. A three-phase search strategy was employed. In phase one, an initial search of MEDLINE (EBSCOhost) was conducted to identify articles related to the topic. This was followed by an analysis of words contained in the titles and abstracts of those articles and the index terms used to describe them. This informed the development of a search strategy tailored to each information source. Phase two involved a second search in which we used all the identified keywords and index terms across each included database. Searches were developed and combined using broad search terms, keywords, and subject headings or thesaurus terms, as applicable to each database. The search strategy was adapted for each database, including all the identified keywords and corresponding index terms. Phase three involved screening the reference lists of all the identified studies selected for critical appraisal to identify additional studies. The databases searched were CINAHL (EBSCOhost), MEDLINE (EBSCOhost), PsycINFO (EBSCOhost), and Igaku Chuo Zasshi (Japan Medical Abstracts Society). The sources of gray literature that we searched were MedNar, ProQuest Dissertations and Theses A&I, ClinicalTrials.gov, and UMIN-CTR. The search strategies for each database are detailed in Table S1. In short, we used the following terms identified by a librarian to conduct the searches: “child”, “school”, “pediatric”, “school-aged”, “adolescent”, “young age”, “food hypersensitivity”, “food allergy”, “anaphylaxis”, “food intolerance”, “food sensitivity”, “emotion”, “adaptation”, “psychological”, “perception”, “psychology”, “behavior”, “phenomena”, “social problems”, “life change events”, “adverse childhood experiences”, “experience”, “peer relationship”, “bully”, “anxiety”, “fear”, “loneliness”, “distress”, “stress”, “developmental challenge”, “identity”, “self-efficacy”, “coping”, “cognitive”, “quality of life”, “stigma”, and “belief”.

### 2.5. Study Selection

The titles and abstracts of all identified articles were screened by two independent reviewers (NN and YM) based on the inclusion criteria. The full text of potentially relevant studies was retrieved and assessed in detail by the same two independent reviewers based on the inclusion criteria. Any disagreements between the reviewers at each stage of the selection process were resolved through discussion or by a third reviewer (SU). The results of this process are presented in a Preferred Reporting Items for Systematic Reviews and Meta-Analyses (PRISMA) flow diagram [20].

### 2.6. Assessment of Methodological Quality

Two reviewers (NN and YM) independently assessed the quality of the included articles by using the Australian JBI Centre for Evidence-based Health Care Qualitative Research Quality Evaluation criteria [21]. Any disagreements that arose between the reviewers were resolved through discussion or by a third reviewer (SU). This evaluation tool consists of 10 items, assessing aspects such as the consistency of the research methodology with its philosophical basis, data collection methods, data analysis methods, results interpretation, the typicality of the research object, the cultural or theoretical positioning of researchers, and ethical review. Each item was assigned a score of 2 for “yes”, 1 for “unclear”, or 0 for “no”. Only articles with a score above 70% were included. If question 8 of the JBI checklist was answered as “no” or “unclear”, authors were asked to provide the data via email, and studies for which no answer to this question were reported were excluded.

### 2.7. Data Extraction and Synthesis

The JBI meta-aggregation approach was employed to extract and synthesize the data [21]. This approach was used to accurately and reliably present the findings of the original authors rather than to reinterpret the studies. Among the methodologies available for the synthesis of qualitative studies, meta-aggregation is the most transparent and widely accepted method for the construction of high-quality systematic reviews of qualitative studies [21].

After careful analysis of the literature, the important information was extracted, including the author, year of publication, country, data collection and analysis methods, phenomena of interest, participant characteristics and numbers, and study results. Each result from each original study was assigned a level of credibility [22]; an outcome was deemed unequivocal when it directly related to what was described in the article. For studies that included participants beyond school age, we extracted only the results that were explicitly supported by raw data attributable to participants aged 6–12 years. For example, in a study of participants aged 6–15 years [23], only the results that were clearly distinguishable as those for the children aged 6–12 years were included.

The qualitative research results were pooled using JBI SUMARI and a meta-aggregation approach [19]. The authors’ analytical interpretation of the results or data were extracted verbatim. The findings were identified through repeated readings of the text and selection of themes from the results section. Subsequently, the survey results were categorized based on their semantic similarity. The categories and related findings were verified via reconsideration in the context of the first study, along with illustrations, and were organized in Microsoft Excel (Microsoft Corp., Redmond, WA, USA). Results with similar meanings were categorized into new groups, which were subsequently integrated to yield novel findings.

These were drafted by one reviewer (NN). Finally, all researchers, including one specialist (IO) with clinical experience in FA medical care, engaged in discussions and confirmed the synthesized results. When the dependability and credibility were assessed, we assumed that the quality of the meta-synthesized evidence was high. Our evaluation was based on three aspects of credibility and five of dependability. The dependability focused on the quality of the original studies included in the analysis, whereas the credibility considered whether the integrated results were consistent with the supporting data.

### 2.8. Assessing Confidence in the Findings

The final synthesized findings were graded according to the ConQual approach to establish confidence in the output of the qualitative research synthesis [22]. The ConQual grades are presented Section 3.4.

## 3. Results

### 3.1. Study Inclusion

**Figure 1:**
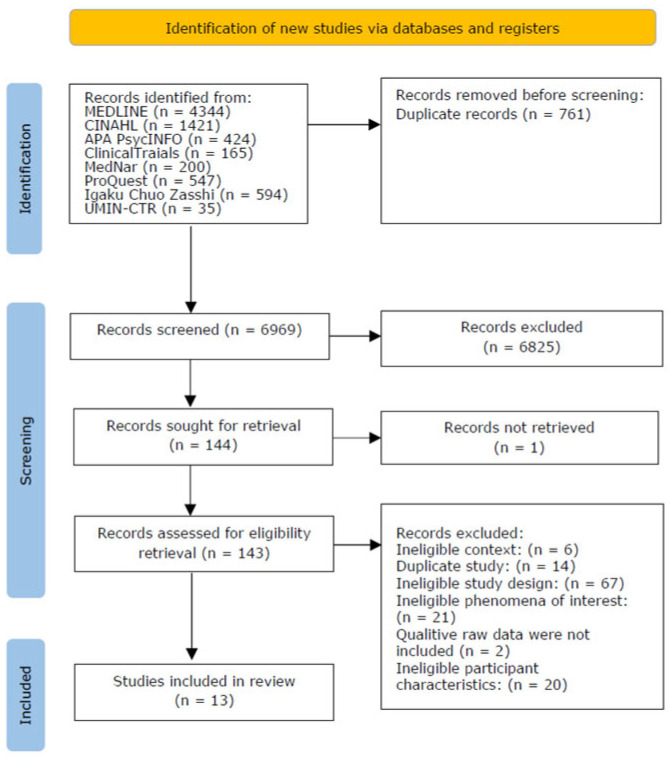
Search results and study selection and inclusion process.

After duplicates were excluded, the initial search produced 6969 articles, of which 15 met the inclusion criteria; these were critically appraised for their methodological quality. One article with a score under 70% was excluded. Two studies were rated unclear in terms of question 8 of the JBI critical appraisal checklist (“Are participants and their voices adequately represented?”) because they did not include the raw qualitative data. The authors of those two articles were contacted to provide additional information; the authors of one article provided the raw data. Overall, 13 qualitative studies were included for review [23,24,25,26,27,28,29,30,31,32,33,34,35]. The PRISMA diagram [20] is presented in Figure 1. The study characteristics are provided in Table 1.

### 3.2. Methodological Quality

Our critical appraisal of the eligible qualitative studies is shown in Appendix A. In terms of the overall quality score, 4 of the 13 included studies met all of the appraisal criteria, 5 met nine of the criteria, and 5 met eight of the criteria. Thus, we obtained sufficient qualitative data on the experiences of children with FAs from each of the 13 studies. However, the cultural/theoretical positioning of the researchers or the influence of the researchers on the results were inadequately described in some of the studies.

### 3.3. Synthesis

A total of 73 findings rated as “unequivocal” were extracted from the 13 studies included in the synthesis. These were aggregated into 13 categories, and then further into three synthesized findings.

### 3.4. Synthesized Finding 1: Living with Fear and Restrictions

Children with FAs live with daily fear, social restrictions, and emotional stress. To ensure their safety and foster self-management skills, their trusted adults must be well-informed about allergy management (Table 2). The first synthesized finding comprised 35 findings from 10 articles, with five categories.

#### 3.4.1. Daily Life Caught in the Fear of Uncertainty About Foods

This category reflects the children’s fears that the foods about which they are uncertain may cause accidental allergen exposure and result in anaphylaxis and even death. The children reported constant fear and anxiety, even regarding the familiar foods in their daily lives. This fear was heightened by past anaphylactic events and the unpredictability of reactions.

*“I’d rather not have it…It’s not fun. And I know I could die from it…I don’t want to die…it doesn’t feel very nice…someone’s choking me…” (age 11)*.

[27]

*“I remember that Mum asked for no nuts, but the ice cream had one in, I could have died.” (age 12)*.

[31]

#### 3.4.2. Living with Vigilance and Avoidance of Allergens

This category reveals that children with FAs always have to be vigilant about safety and risk in order to avoid allergens. To stay safe, the children adhere to strict rules: not to eat any food about which they are unsure, avoiding shared food, reading labels, and following hygiene measures. Children with FAs often take their own meals along wherever they go and avoid unfamiliar environments, leading to hyper-vigilance.

*“The most annoying part, annoying to handle, is having to read labels and ask everywhere I go ‘do these have nuts?’” (age 11)*.

[24]

*“if you just stay with the people that know you, you will be ok.” (age 11)*.

[23]

#### 3.4.3. Living with Daily Limitations

This category relates to how the social and school activities of children were limited owing to FAs. The children had to avoid restaurants, birthday parties, and school events. Although some normalized these limitations, others expressed frustration and sadness.

*“For birthday parties, she [my mom] doesn’t let me go because she’s worried that the cake will have peanuts or something. Or maybe like events that some of my friends are going to, she wouldn’t let me go unless she came with me because she’s worried that I would end up eating something or playing around and mess up.” (age 10)*.

[24]

*“I’ve kind of gotten used to it. So, it probably taught me some good lessons, I don’t know, like, I feel like I’m more—maybe not as soft as the average kid just because I’ve had to go through disappointment and stuff like that. But it’s not a big thing anymore because I’m used to it. So, I can just be—it’s just my normal routine at this point.” (age 11)*.

[27]

#### 3.4.4. Self-Management with Support

This category includes findings relating to the importance of support from trusted adults to increase the children’s confidence in managing their FAs. As children grow, they transition from parental oversight to self-management. Support from teachers, school staff, parents, and structured environments enhances children’s confidence and autonomy. Teachers were also mentioned as assisting with the children’s safety through proactive measures.

*“Usually, I ask (the teachers) about all the food before I do anything with it. I’m fairly confident. I asked about what the food was, like if there was anything in it, and sometimes I know perfectly about the food because they [teachers] tell me beforehand about the food. It made me feel more comfortable when they [teachers] told me beforehand about the food.” (age 9)*.

[29]

*“Momma usually does it. She tells the teacher at the beginning of the school year. Momma fills out all the paperwork. She tells them that I have a peanut allergy.” (age 9)*.

[29]

#### 3.4.5. Burden of Management and Coping

In this category, the children reported their adoption of avoidance or denial as a coping strategy for the emotional and practical burdens of FA management. The children hesitated to use emergency medication or felt embarrassed about their condition.

*“I don’t like showing [the auto-injector], so I try to hide it so I look just normal like.” (age 10)*.

[31]

### 3.5. Synthesized Finding 2: Isolation and Empathy in Peer Relationships

Children with FAs experience isolation, teasing, and exclusion from their peers; peer communication can foster understanding and acceptance. To address this, schools must promote peer empathy, provide allergy education, and build inclusive environments that empower children to express their needs safely (Table 3). The second synthesized finding comprised 24 findings obtained from eight articles, with five categories.

#### 3.5.1. Feeling Isolated or Different from Peers

This category revealed that children with FAs often feel different from or excluded by their peers. When the children were unable to do the same things as their classmates, they experienced loneliness, frustration, and a desire to be normal.

*“I hated sitting by myself at lunchtime, or having to get a ‘special’ treat…. like there’s something wrong with me.” (age 12)*.

[31]

*“When we made bread at school, I forgot to check, so I could not eat together with everyone. I was disappointed.” (age 8)*.

[34]

#### 3.5.2. Lack of Understanding Among Classmates

This category included children’s reports that their classmates lacked awareness and empathy regarding their FAs. This led to emotional pain, frustration, and a sense of being disregarded or unsafe.

*“And once I get to school, the real challenges start because there, they don’t care about me as much as my family does. And they don’t know as much as I do…once this person had tree nuts and they were eating them right next to me. So I told them to put them away or throw them away… or move away to another table or something—but they said no. They wouldn’t move away or put them away.” (age 12)*.

[27]

#### 3.5.3. Bullying and Teasing

Some children are the targets of bullying or even dangerous behaviors at times, such as being threatened with allergens. These incidents contribute to fear, shame, and a reluctance to disclose their condition.

*“It only happened last year with the boys throwing peanuts. It never happened in K5. Only in 1st grade. I felt mad. Then they were trying to throw peanuts into my ice cream.” (age 9)*.

[29]

*“I was teased by my classmate, who said ‘Your lunch box smells bad’.” (age 9)*.

[34]

#### 3.5.4. Strategies to Manage Identity/Emotions

This category concerns children’s strategies to cope with negative situations. To cope with social challenges, children adopt various strategies: ignoring negative comments, sharing their feelings with their parents, or trying to blend in. These efforts reflect resilience and emotional self-regulation.

*“I sometimes shoot back but I mostly put up with it. There are friends who say nice things like ‘yours looks tasty.’” (age 9)*.

[34]

#### 3.5.5. Support and Acceptance Through Communicating with Peers

In contrast to the negative attitudes of classmates, this category pertains to support from peers and the importance of friendships. These children decided to disclose their FAs to others and advocate for themselves. The children mentioned the positivity of acceptance through communicating their FAs to others.

*“I feel very confident! Well, umm some of my friends, they don’t usually make fun of me for having a peanut allergy. They don’t say, "Oh, you are missing out on a lot." I even have a friend who doesn’t like peanuts at all, so he gets to sit by me every day because he doesn’t like peanuts.” (age 9)*.

[29]

### 3.6. Synthesized Finding 3: Challenges of and Hopes for Oral Immunotherapy or Allergen Reintroduction

Motivated by curiosity and personal goals, some children engage in oral immunotherapy (OIT) or allergen reintroduction, leading to meaningful outcomes, such as increased food choices, social inclusion, and reduced anxiety. At the same time, they face emotional and physical burdens, highlighting the need for safety-focused, informed, and supported care that considers both the benefits and burdens (Table 4). The third synthesized finding comprised 14 findings obtained from three studies, with three categories.

#### 3.6.1. Motivation and Participation to Overcome FAs

This category shows the sense of agency among children with FAs in terms of their treatment. These children tried OIT or reintroduced foods with the hopes of eating more freely and feeling connected to their peers. The children made choices about which foods to try and actively participated in discussions with their parents and doctors.

*“I eat bread that has a little bit of milk. I also eat snacks that have only a tiny bit of milk.” (Interviewer: “Right. Does your mom choose those foods?”) “Erm, she buys whatever I want to eat, and I eat it.” (Interviewer: “So, you go to the shop with her?”) “Yes.” (Interviewer: “Does she say, for example, ‘shall we try this food because it’s got a little bit of milk?’) “I ask if I can eat this food, and if he/she says yes, I eat it.” (Interviewer: “Is that something you ask your doctor at your visit? Do you tell the doctor which food you want to try?”) “Yeah.” (age 10)*.

[35]

#### 3.6.2. Joy and Expansion of Daily Life Owing to Reintroduction of Foods Eliminated from Diet

In this category, the children reported that the successful reintroduction of allergens brought positive changes to their lives. They gained access to a wider variety of foods; could participate more fully in social settings, such as school lunches and parties; and experienced reduced anxiety. The children felt more normal and connected to their peers.

*“I have a much larger variety of stuff to eat… You’re allergic to one less thing, and one less thing is a lot for allergies!” (age 9)*.

[26]

#### 3.6.3. Burden to Try OIT or Reintroduce Allergens

Despite the benefits, this category shows that children are also faced with emotional and physical burdens. These included the fear of allergic reactions, discomfort with medical procedures, and pressure to eat foods that they disliked.

*“I felt like I wouldn’t be able to breathe if I ate it, that I would have to use the big shot… the EpiPen” (aged 10)*.

[26]

*“I don’t like eggs themselves, but my mom tells me to eat them. I could eat an egg if it is sweetened, but she tells me to eat it as it is.” (age 11)*.

[33]

### 3.7. Quality Appraisal of Synthesized Findings

The quality appraisal of the synthesized findings is presented in Table 5. Overall, each synthesized finding has a moderate ConQual score.

## 4. Discussion

In this qualitative systematic review, we explored the experiences of school-age children with FAs by listening to their voices. Three integrated findings were developed in an attempt to understand the psychosocial experiences of this group. We restructured these consolidated findings to highlight guidelines for action on which to base recommendations. Below, we present a series of statements in which the integrated findings are expressed so as to accurately convey their meanings.

### 4.1. Living with Fear and Limitations

“Children with FAs live with daily fear, social restrictions, and emotional stress. To ensure their safety and foster self-management skills, trusted adults must be well-informed about allergy management.” The first synthesized finding highlights the intense fear and uncertainty that children face regarding allergen exposure. For many, the potential of a life-threatening reaction is not abstract but a lived experience. The children described symptoms, such as choking, swelling, and fear of death [23,27], vividly illustrating the emotional toll of living with FAs. This fear is worsened by the unpredictability of reactions—even previously safe foods can suddenly cause issues [31], leading to anxiety and confusion. As a result, the children often adopt restrictive behaviors, avoiding school lunches, social events, and unfamiliar foods [32,34]. These limitations are deeply social and emotional, often resulting in isolation and a sense of being different. This psychological burden is further substantiated by the findings of LeBovidge et al. [36], who demonstrated that children aged 8 years and older with FAs exhibit elevated levels of anxiety-related coping behaviors and symptoms of separation anxiety. They also revealed a correlation between negative attitudes toward FAs and increased symptoms of anxiety, depression, and social stress [36]. These psychological impacts underscore the importance of recognizing and addressing the mental health needs of children with FAs alongside their physical safety.

To manage these challenges, children depend on the support of trusted adults. Teachers, parents, and caregivers are crucial in ensuring safety and enabling participation. The children reported that they feel safer when their teachers are trained in emergency responses and when their parents help them to make food choices [29,34]. However, while the need for the training of school staff in FA management has been emphasized [37,38], they are currently ill-prepared [39,40,41]. Moreover, gaps in the public understanding of FAs underscore the critical role of advocacy for raising awareness and driving systemic change [42]. This gap is also reflected in the GA^2^LEN (the Global Allergy and Asthma European Network) and EFA (the European Federation of Allergy and Airways Diseases Patients’ Associations) consensus statement, which highlights that many schools remain underprepared to manage FAs and anaphylaxis, reinforcing the need for standardized training and inclusive practices [43]. Thus, healthcare providers must educate both children and the adults around them, including school staff and families, to balance safety with children’s growing independence. As children mature, they gradually take on more responsibility, but this shift must be supported by informed, empathetic adults. Therefore, healthcare providers must not only educate children about self-management but also equip the adults around them with accurate, practical knowledge about allergy care. This includes the training of school staff, informing the children’s peers, and supporting families.

### 4.2. Building Inclusive Peer Relationships in School Settings

“Children with FAs experience isolation, teasing, and exclusion from peers; peer communication can foster understanding and acceptance. To address this, schools must promote peer empathy, provide allergy education, and build inclusive environments that empower children to express their needs safely.” This second synthesized finding emphasizes the importance of peer relationships in the lives of school-age children with FAs.

The children reported being excluded during lunch or group activities and treated unfairly owing to their dietary restrictions [27,31]. Some experienced bullying, including verbal taunts and dangerous actions, such as being hit with allergens [29,34]. Those experiences often led to anxiety, self-isolation, and a reluctance to disclose their condition, especially as they became more aware of peer judgment. A previous systematic review revealed that 17–60% of children with FAs are bullied, mostly in schools, resulting in long-term psychological effects, such as depression and social withdrawal, impacting both the children and their families [7]. FA-related bullying is a serious problem that society needs to address.

However, peer relationships also have the potential to have positive effects. Some of the children felt supported by friends who respected their allergies and helped them to engage safely [29]. Such inclusive moments fostered a sense of belonging. Some children also actively managed their social identity—by selectively disclosing their allergy, advocating for themselves, or seeking adult support [27,34]—demonstrating resilience and highlighting the need for systemic support. This aligns with the GA^2^LEN and EFA consensus statement, which emphasizes the importance of allergy education and inclusive school policies to reduce stigma and foster peer empathy [43]. To build inclusive environments, schools must promote allergy education and empathy. Although ensuring that schools have trained and cooperative school personnel is important, they may be ill-prepared to manage the psychological issues associated with FAs [40]. This gap underscores the need for comprehensive training that includes both the medical and emotional aspects of FAs. This could include regular in-service training for teachers on allergy management, classroom activities that foster peer understanding (e.g., role-play and story-based discussions), and the integration of FA awareness into health education curricula. One example is the Be a PAL^®^ (Protect A Life) program by Food Allergy Research & Education (FARE), which provides age-appropriate materials and interactive activities to teach students how to support peers with FAs [44]. Teachers are key players in shaping classroom culture and normalizing accommodations. Empowering children with FAs to express their needs—and ensuring that their peers understand and respect them—can shift the social dynamic from exclusion to support. As peer relationships can either intensify or ease the burden of FAs, schools and communities must foster understanding, acceptance, and safety.

### 4.3. Children’s Agency and Support for Their Needs During Oral Immunotherapy and Food Reintroduction

“Motivated by curiosity and personal goals, children engage in OIT or allergen reintroduction, leading to meaningful outcomes such as increased food choices, social inclusion, and reduced anxiety. At the same time, they face emotional and physical burdens, highlighting the need for safety-focused, informed, supported care that considers both benefits and burdens.” This third synthesized finding marks a shift in FA management. Whereas strict avoidance was once the norm, current EAACI guidelines recommend periodic reassessment and oral food challenges to explore reintroduction [45]. Management has evolved from observation to intervention, including the use of OIT and other treatments [3].

The children’s accounts in this review revealed that they were active participants in these interventions. Motivated by personal goals—such as eating the same foods as their peers or enjoying treats—they expressed curiosity and a desire to try new foods [33,35]. The benefits were notable: increased dietary variety, participation in social events, and reduced anxiety [26,33]. Being able to enjoy ice cream at a party or worry less in public spaces substantially improved the quality of life of some children with FAs [26]. Support for this finding is provided by other studies that have showed that OIT can significantly improve the quality of life of children with FAs by reducing anxiety and increasing dietary freedom [46,47]. However, during the course of OIT, some children experience a temporary decline in their quality of life owing to physical discomfort, emotional stress, and fear of reactions [48]. In the studies that we evaluated, the children reported fearing allergic reactions, feeling discomfort with the medical procedures [26], and feeling pressure to eat foods that they disliked [33]. These burdens emphasize the need for care that is both medically and emotionally supportive. The children also showed agency—expressing preferences, making decisions, and communicating with their caregivers and providers [35]. These findings suggest that clinicians should actively involve children in shared decision-making processes, using developmentally appropriate communication tools (e.g., visual aids or simplified explanations), and allow them to express hesitation or refusal without feeling pressured. To ensure that the benefits of OIT and food reintroduction are realized while the burdens are minimized, collaboration between the healthcare teams—including physicians and allergy nurses—and families is essential, creating safe, informed, and supportive environments.

### 4.4. Strengths and Limitations

This integrative review was focused exclusively on the experiences of school-age children living with FAs to enable the voice of this vulnerable population to be heard. Based on the ConQual scores [22], all three synthesized findings were evaluated as moderate quality. Some studies did not adequately address the description of the cultural/theoretical positioning of the researchers (Critical Appraisal Checklist question 6 [22]) or the potential influence of the researchers on the research process (question 7 [22]).

OIT and food reintroduction are relatively recent treatment approaches, and the synthesized findings related to these interventions are both novel and significant. However, these findings were derived from only three studies. To elucidate the outcomes and burdens associated with these treatments, further research is needed—particularly through the synthesis of additional evidence. Furthermore, the majority of the included studies were conducted in high-income countries, such as the United States, the United Kingdom, and Japan. Thus, the findings may not fully capture the experiences of children with FAs living in low- and middle-income countries, where access to healthcare, allergy awareness, and social attitudes may differ considerably. Therefore, the recommendations from this review should be interpreted with caution.

## 5. Conclusions

In this qualitative systematic review, we explored the lived experiences of school-age children with FAs, synthesizing their voices into three key findings. First, these children live with constant fear over allergen exposure, leading to emotional stress and restricted social participation. To manage these challenges, their trusted adults must be well-informed and provide consistent, supportive guidance. The fostering of self-management in safe environments is essential to reduce anxiety and promote autonomy. Second, peer dynamics substantially shaped these children’s experiences. Many reported isolation, bullying, teasing, or exclusion owing to their FAs. However, when their peers were empathetic and informed, they could become strong allies. Schools play a crucial role in the promotion of peer empathy, the provision of allergy education, and the establishment of inclusive environments where children feel safe to express their needs and engage socially. Third, some children pursued OIT or allergen reintroduction, driven by curiosity and the desire for normalcy. Those interventions led to improvements, such as increased food variety, reduced anxiety, and greater social engagement. However, treatment also brought physical and emotional burdens. Some children showed agency by engaging in their care and voicing their preferences. This highlights the need for care that respects children’s voices and supports their active role in decision-making. In summary, children with FAs are not merely managing a condition—they are growing within complex social and emotional contexts. Therefore, holistic, child-centered support across the home, school, and healthcare settings is essential to help them thrive.

## Figures and Tables

**Table 1:** Study characteristics.

Study	Methods for Data Collection and Analysis	Country	Phenomena of Interest	Sample Size (Participants’ Ages)	Results (Findings Supported by Raw Data of School-Age Children)
DunnGalvin et al., 2009 [23]	Data collection: focus group meetings Data analysis: grounded theory	Ireland	Psychosocial development related to FAs in children.	62 children (6–15 years)	Meanings of food Autonomy and control Peer relationships Risk and safety Self/identity Coping strategies
Herbert et al., 2023 [24]	Data collection: separate qualitative interviews Data analysis: grounded theory	USA	Biopsychosocial experiences regarding FA-related psychosocial functioning and adherence.	26 adolescents (9–14 years)	FA is a chronic burden that affects daily life. Families experience anxiety about FAs. FA families feel the need to be prepared. FA families frequently advocate for their needs.
Monks et al., 2010 [25]	Data collection: semi-structured interviews Data analysis: thematic approach	England	Practical challenges that teenagers with FAs experience.	18 teenagers (11–18 years)	Allergen avoidance Education about food allergies
LeBovidge et al., 2014 [26]	Data collection: semi-structured interviews Data Analysis: grounded theory	USA	Psychological impact of OIT on children and families.	10 children (9–18 years)	Increased dietary options Inclusion in social situations Decreased anxiety Difficulty with injections Anxiety about ingesting milk Allergic reactions
Martinez et al., 2024 [27]	Data collection: individual semi-structured interviews Data analysis: interpretive phenomenological analysis	USA	Lived experiences of adolescents living with FAs and psychosocial impact of FAs on their daily lives.	4 children (11–12 years) and 10 adolescents (13–14 years)	Living with restraints: a way of life Managing exposure Experiencing stigma Experiencing unawareness
Fenton et al., 2011 [28]	Data collection: drawing and explaining a picture, in-depth face-to-face interviews Data analysis: integrated analytic framework (reflexive analysis, thematic analysis, and depth analysis)	Canada	Perceptions and experiences of Ontario students with severe FAs, focusing on their views of school as a safe place and the psychosocial stresses associated with risk.	10 children (8–12 years) and 10 adolescents (13–18 years)	Social and environmental barriers to safety Coping strategies Emotional burden of responsibility
Koel, 2017 [29]	Data collection: draw-and-tell exercises, self-efficacy scale, and child-centered interviews Data analysis: phenomenological study	USA	Development of self-efficacy in children with PAs in educational settings.	6 children (7–10 years)	Self-management Communication Emotional experiences Self-management (vigilance and safety) Bullying Advocation Proactivity Trust Facing change Safety Peer acceptance Importance of friendships
DunnGalvin et al., 2020 [30]	Data collection: semi-structured interviews Data analysis: thematic analysis	UK, France, Germany	Strategies used to cope with and manage PAs and the impact of these strategies on the quality of life of children, teenagers, and caregivers.	24 children (8–12 years), 39 teenagers (13–17 years), and 44 caregivers	Daily monitoring/vigilance/avoidance Emotional impact Social and school activities Practicalities and planning Communicating
DunnGalvin et al., 2018 [31]	Data collection: focus groups using semi-structured interviews Data analysis: deductive thematic analysis	Australia, Ireland, Italy, UK, USA	Usefulness of the FA-specific developmental model (FACE questionnaire) for explaining emotions and coping styles among children and young adults, including mediators of coping style.	274 children and young adults (6–23 years) and 119 parents	Living with uncertainty Coping and management Living with the rules Living with difference
Byrne, 2022 [32]	Data collection: semi-structured interviews Data analysis: general inductive approach	USA	Impact of severe FA reactions and post-traumatic symptoms on children and caregivers.	6 children (9–15 years) and their mothers; included 4 children aged 9–11 years	Heightened vigilance in response to threat as result of child’s anaphylactic event. The impact of food allergy on general day-to-day functioning. Increased confidence in managing child’s food allergy as result of anaphylactic event.
Saito et al., 2017 [33]	Data collection: semi-structured interviews Data analysis: qualitative inductive analysis	Japan	Experiences and feelings toward the process of reintroducing foods that had been eliminated from the children’s diets owing to FAs.	6 children (9–14 years) and their mothers; included 5 children aged 9–11 years	Reintroduction of foods after an elimination diet. More variety in meals. Thoughts on reintroduction of foods after an elimination diet. Experience of foods broadened. Thoughts when foods reintroduced after elimination diet. Feeling of being able to eat the same thing as friends.
Yamada et al., 2016 [34]	Data collection: semi-structured interviews Data analysis: qualitative induction	Japan	Problems faced by school-aged children with FAs during everyday school life and the approaches taken to overcome these problems.	9 children (7–12 years) and 9 parents	Teased and bullied by classmates. Worried if classmates will understand. Unable to do the same things as classmates. Fear of accidental exposure. Hesitation in injecting EpiPen. Share feelings with parents. Withstanding uncomfortable situations. Pay careful attention to school lunches. Make an effort to reduce feelings of isolation. Trust teachers at school and seek help.
Yuguchi et al., 2021 [35]	Data collection: semi-structured interviews Data analysis: qualitative inductive analysis	Japan	Actual self-care situation of school students with FAs and parents’ involvement in expanding children’s self-care.	14 children (6–11 years)	Preventing accidental exposure. Management of anaphylaxis. Relationships with people. Thoughts on present and future. Participation in disease understanding and treatment.

FA, food allergy; FACE, Food Allergy Coping and Emotions; OIT, oral immunotherapy; PA, peanut allergy.

**Table 2:** Synthesized finding 1: Living with fear and restrictions.

Children With FAS Live Everyday With Fear, Social Restrictions, and Emotional Stress. To Ensure Their Safety and Foster Self-Management Skills, Trusted Adults in Their Lives Must Be Well-Informed About Allergy Management.	
Categories	Authors’ Findings
Daily life caught in the fear of uncertainty about foods	Fear and uncertainty (U) Living with uncertainty (U) Fear of accidental exposure (U) Meanings of food (U) Emotional impact (U) Autonomy, control, and self-efficacy (U) Heightened vigilance in response to threat as result of child’s anaphylactic event (U)
Living with vigilance and avoidance of allergens	Daily monitoring/vigilance/avoidance (U) Allergen avoidance (U) Vigilance (U) Self-management (vigilance and safety) (U) Avoidance (U) Risk and safety (U) Safety (U) FA is a chronic burden that affects daily life (U) Pay careful attention to school lunches (U)
Living with daily limitations	Limitations (U) Living with the rules (U) Social and school activities (U) Strategies to manage risk/emotions (U) Way of life (U) Emotional burden of responsibility (U) The impact of FA on general day-to-day functioning (U) Families experience anxiety about FAs (U)
Self-management with support	Self-management (U) Trust (U) Trust teachers at school and seek help (U) FA families feel the need to be prepared (U) Coping strategies (U) Increased confidence in managing child’s FA as result of anaphylactic event (U) Proactivity (U)
Burden of management and coping	Facing change (U) Management of anaphylaxis (U) Hesitation in injecting EpiPen (U) Coping and management (U)

FA, food allergy; U, unequivocal.

**Table 3:** Synthesized finding 2: Isolation and empathy in peer relationships.

Children With FAS Experience Isolation, Teasing, and Exclusion From Peers, While Peer Communication Can Foster Understanding and Acceptance. To Address This, Schools Must Promote Peer Empathy, Provide Allergy Education, and Build Inclusive Environments That Empower Children to Express Their Needs Safely.	
Categories	Authors’ Findings
Feeling isolated or different from peers	Living with difference (U)
	Perceived as different (U)
	Sense of identity (U)
	Sense of loneliness (U)
	Emotional experiences (U)
	Unable to do the same things as classmates (U)
Lack of understanding among classmates	Lack of understanding (U)
	Worried if classmates will understand (U)
	Dismissive (U)
Bullying and teasing	Bullying (U)
	Teased and bullied by classmates (U)
	Peer relationships (U)
Strategies to manage identity/emotions	Strategies to manage identity/emotions (U)
	Make an effort to reduce feelings of isolation (U)
	Withstanding uncomfortable situations (U)
	Share feelings with parents (U)
Support and acceptance through communicating with peers	Peer acceptance (U)
	Advocation (U)
	Making decisions (U)
	FA families frequently advocate for their needs (U)
	Importance of friendship (U)
	Communication (U)
	Education about FAs (U)
	Gain understanding of classmates (U)

FA, food allergy; U, unequivocal.

**Table 4:** Synthesized finding 3: Challenges of and hopes for oral immunotherapy or allergen reintroduction.

Motivated by Curiosity and Personal Goals, Children Engaged in OIT or Allergen Reintroduction, Leading to Meaningful Outcomes, Such as Increased Food Choices, Social Inclusion, and Reduced Anxiety. At the Same Time, They Faced Emotional and Physical Burdens, Highlighting the Need for Safety-Focused, Informed, and Supported Care That Considers Both the Benefits and Burdens.	
Categories	Authors’ Findings
Motivation and participation to overcome FAs	Thoughts on reintroduction of foods after an elimination diet (U)
	Thoughts when foods were reintroduced after elimination diet (U)
	Thoughts on present and future (U)
	Participation in disease understanding and treatment (U)
Joy and expansion of daily life owing to reintroduction of foods eliminated from diet	Increased dietary options (U)
	Broadened experience of foods (U)
	Inclusion in social situations (U)
	More variety in meals (U)
	Decreased anxiety (U)
	Feeling of being able to eat the same things as friends (U)
Burden to try OIT or reintroduce allergens	Anxiety about ingesting milk (U)
	Being encouraged to reintroduce foods after an elimination diet (U)
	Injections (omalizumab, blood draws) (U)
	Allergic reactions (U)

FA, food allergy; OIT, oral immunotherapy; U, unequivocal.

**Table 5:** Quality appraisal of synthesized findings.

Synthesized Finding	Dependability	Credibility	ConQual Score
Children with FAs live with daily fear, social restrictions, and emotional stress. To ensure their safety and foster self-management skills, trusted adults in their lives must be well-informed about allergy management.	Downgrade 1 level	Not downgraded	Moderate
Children with FAs experience isolation, teasing, and exclusion from their peers, while peer communication can foster understanding and acceptance. To address this, schools must promote peer empathy, provide allergy education, and build inclusive environments that empower children to express their needs safely.	Downgrade 1 level	Not downgraded	Moderate
Motivated by curiosity and personal goals, children engage in OIT or allergen reintroduction, leading to meaningful outcomes, such as increased food choices, social inclusion, and reduced anxiety. At the same time, they face emotional and physical burdens, highlighting the need for safety-focused, informed, and supported care that considers both benefits and burdens.	Downgrade 1 level	Not downgraded	Moderate

FA, food allergy; OIT, oral immunotherapy.

## Data Availability

The raw data supporting the conclusions of this article will be made available by the authors on request.

## Supplementary Materials

The following supporting information can be downloaded at https://doi.org/10.5281/zenodo.16560918, Table S1: Search strategy.

## Abbreviations

The following abbreviations are used in this manuscript:

EAACI	European Academy of Allergy and Clinical Immunology
FA	Food Allergy
JBI	Joanna Briggs Institute
OIT	Oral Immunotherapy
PRISMA	Preferred Reporting Items for Systematic Reviews and Meta-Analyses

## Appendix A

**Table A1:** Critical appraisal of the eligible qualitative studies.

Citation	Q1	Q2	Q3	Q4	Q5	Q6	Q7	Q8	Q9	Q10
DunnGalvin et al., 2009 [23]	Y	N	Y	Y	Y	Y	Y	Y	Y	U
Herbert et al., 2023 [24]	Y	Y	Y	Y	Y	Y	Y	Y	Y	Y
Monks et al., 2010 [25]	Y	Y	Y	Y	Y	Y	Y	Y	Y	Y
LeBovidge et al., 2014 [26]	Y	Y	Y	Y	Y	Y	Y	Y	Y	Y
Martinez, 2020 [27]	Y	Y	Y	Y	Y	Y	Y	Y	Y	Y
Fenton et al., 2011 [28]	Y	Y	Y	Y	Y	N	Y	Y	Y	Y
Koel, 2017 [29]	Y	Y	Y	Y	Y	N	Y	Y	Y	Y
DunnGalvin et al., 2020 [30]	Y	Y	Y	Y	Y	Y	Y	Y	Y	Y
DunnGalvin et al., 2018 [31]	Y	Y	N	Y	Y	Y	Y	Y	Y	Y
Byrne, 2022 [32]	Y	Y	Y	Y	Y	Y	Y	Y	Y	Y
Saito et al., 2017 [33]	Y	Y	Y	Y	Y	N	N	Y	Y	Y
Yamada et al., 2016 [34]	Y	Y	Y	Y	Y	N	N	Y	Y	Y
Yuguchi et al., 2021 [35]	Y	Y	Y	Y	Y	Y	N	U	Y	Y

Y, yes; N, no; U, unclear. Joanna Briggs Institute critical appraisal checklist for qualitative research. Q1 = Is there congruity between the stated philosophical perspective and the research methodology? Q2 = Is there congruity between the research methodology and the research question or objectives? Q3 = Is there congruity between the research methodology and the methods used to collect data? Q4 = Is there congruity between the research methodology and the representation and analysis of data? Q5 = Is there congruity between the research methodology and the interpretation of results? Q6 = Is there a statement locating the researcher culturally or theoretically? Q7 = Is the influence of the researcher on the research, and vice versa, addressed? Q8 = Are participants and their voices adequately represented? Q9 = Is the research ethical according to current criteria or, for recent studies, is there evidence of ethical approval by an appropriate body? Q10 = Do the conclusions drawn in the research report flow from the analysis, or interpretation, of the data?
